# Hydrothermal Treatment of Arsenic Sulfide Residues from Arsenic-Bearing Acid Wastewater

**DOI:** 10.3390/ijerph15091863

**Published:** 2018-08-28

**Authors:** Liwei Yao, Xiaobo Min, Hui Xu, Yong Ke, Yanjie Liang, Kang Yang

**Affiliations:** 1 School of Metallurgy and Environment, Central South University, Changsha 410083, China; yaoliwei0125@126.com (L.Y.); Xuhui940303@163.com (H.X.); Liangyanjie2015@csu.edu.cn (Y.L.); 2 Chinese National Engineering Research Center for Control & Treatment of Heavy Metal Pollution, Changsha 410083, China; 3 School of Minerals Processing and Bioengineering, Central South University, Changsha 410083, China; csuyangkang@163.com

**Keywords:** arsenic sulfide residue, hydrothermal treatment, dehydration, volume reduction, stabilization/solidification, arsenic-bearing wastewater

## Abstract

Arsenic sulfide residue (ASR), a by-product from the treatment of arsenic-bearing acidic wastewater, is abundantly generated but not properly disposed of in China. The utilization of such high-content arsenic waste residue is limited by the market. The traditional methods of stabilization/solidification (S/S) by lime cement or iron salt have a large mass/volume addition, high dumping cost and secondary pollution risk. In this paper, hydrothermal technology was used to treat three kinds of ASRs obtained from different smelters to minimize waste. The leaching toxicity and chemical speciation of the generated products was also evaluated by TCLP and BCR analyses. It was found that the hydrothermal treatment could greatly reduce the volume and moisture content of the ASRs. TCLP tests showed that the leachability of arsenic and heavy metals significantly decreased after the treatment. According to the BCR analysis, most of the unstable As, Cd and Cr transformed into a residual fraction. Finally, XRD, SEM, Raman and XPS techniques were carried out to reveal the mechanism. As a result, hydrothermal treatment can efficiently achieve the dehydration, volume reduction and stabilization/solidification of ASRs.

## 1. Introduction

Arsenic-containing waste is widely produced due to anthropogenic activities [1,2,3,4]. It is universally believed that the extractive process of non-ferrous metals is one of the most important sources of arsenic [5,6]. In the smelting of copper or lead-zinc ore, larger amounts of acidic wastewater are discharged when gaseous emission containing SO_2_, As, Cu, Pb, Zn and other impurities are washed with diluted acid [7]. Moreover, wasted electrolyte with a high concentration of arsenic is produced abundantly in copper and lead-zinc electrolyte processes [8,9]. At present, there are many treatment methods for arsenic-containing wastewater, such as precipitation, adsorption, and biological methods [10,11,12,13,14]. However, such wastewater is usually treated with sodium sulfide, sodium hydrosulfide or hydrogen sulfide to precipitate arsenic, and the deposition of arsenic sulfide residue (ASR) is then engendered [15,16]. It is estimated that hundreds of thousands of tons of ASR are generated annually in China. As a typical arsenic-containing waste, ASR will lead to serious environmental pollution if disposed of improperly.

In the past decades, many efforts have been devoted to this subject. One of the directions is to reclaim arsenic from ASR. A vacuum method was proposed to recover elemental sulfur, arsenic trioxide and arsenic sulfide from ASR in a step by step fashion [17]. In addition, a technique in which the ASR was reacted with CuSO_4_ was developed for the recovery of As_2_O_3_ [7]. Despite these achievements, the application of these technologies is limited because the market for arsenic is shrinking. Therefore, stabilization/solidification (S/S) has drawn the attention of the related researchers. The S/S process was recognized as “the best demonstrated available technology” for the disposal of solid waste by the US Environmental Protection Agency (USEPA). It is proven that the leaching concentrations of arsenic and other heavy metals in the waste could be effectively decreased after it is stabilized/solidified with lime, cement, or smelting slag [18,19,20,21]. For the case of ASR, a significant drop in the mobility of arsenic was also confirmed after S/S [22,23]. However, it could be noted that, because of the involvement of the addition of other chemicals, the volume and/or weight would be increased, which could ultimately lead to an uneconomic disposal.

Recent studies have shown that hydrothermal treatment can effectively achieve the stabilization of hazardous waste but also realize the dehydration of the waste and hence reduce the volume of waste [24]. Vinals et al. [25] found that arsenic in calcium arsenate waste from a copper smelter can be stabilized by hydrothermal treatment, generating the precipitation as arsenical natroalunite which is effective for long-term storage. Qiu et al. [26] indicated that hydrothermal treatment assisted by microwave heating is a feasible approach for the solidification of heavy metals in fly ash. Our previous studies [27,28,29,30] determined that stabilization of heavy-metal-containing neutralization sludge and dehydration of calcium sulfate dihydrate and calcium sulfate hemihydrate in the neutralization sludge occurred during hydrothermal treatment.

To the best of our knowledge, no study concerning the stabilization, dehydration and volume reduction of ASR by hydrothermal treatment has been reported. Therefore, in this research, ASRs collected from various smelting companies were subjected to the hydrothermal treatment. It is expected that the leachability of arsenic and the moisture and volume in the ASRs will be greatly decreased.

## 2. Materials and Method

### 2.1. Materials

Arsenic sulfide residues used in the experiments were obtained from three nonferrous metal smelting companies located in Hubei, Shandong and Anhui, China, which were marked as HB-ASR, SD-ASR and AH-ASR, respectively. The ASRs were generated during the treatment of acidic wastewater with H_2_S. The collected ASRs were stored in the companies for several months or years. The raw pH of ASR is measured in water suspensions with a mass ratio of ASR to solution of 1:10. The selected physicochemical properties of the raw ASRs are presented in Table 1.

### 2.2. Experimental Procedure

The collected ASR (10 ± 0.01 g) without drying was added into a 20 mL autoclave, which was heated to different temperature and then maintained for various periods. Unless otherwise specified, the hydrothermal treatment experimental conditions of HB-ASR, SD-ASR and AH-ASR were performed at 160 °C for 4 h. Thereafter, the autoclave was cooled to room temperature under tap water. Finally, the resulting products were filtered with 1 μm filter papers. The treated residues were collected, washed with 10 mL deionized water, and then dried at 60 °C overnight in a vacuum oven. To evaluate the stability of the filter residue, the toxicity characteristic leaching procedure (TCLP) was performed. The filtered hydrothermal fluid contained high concentrations of heavy metals, and the concentration of heavy metals were determined using ICE-AES.

### 2.3. Analysis

#### 2.3.1. Determination of the Dehydration Ratio and Volume Reduction Ratio

Moisture content was measured by drying the samples at 60 °C under vacuum to a constant weight (±0.01 g). The dehydration ratio was then calculated according to Equation (1). The volume of samples before and after hydrothermal treatment was measured by the method of water displacement [31]. The volume reduction ratio can then be calculated according to Equation (2):

(1) $$Dehydration\;ratio=\frac{M\times \varphi -M^{\prime }\times \varphi^{\prime }}{M\times \varphi }\times 100\%$$

(2) $$Volume\;reduction\;ratio=\frac{V-V^{\prime }}{V}\times 100\%$$

where *M* (g) is the raw weight of the ASR; *ϕ* (%) is the raw moisture of the ASR; $M^{\prime }$ (g) is weight of the treated ASR; $\varphi^{\prime }$ (%) is the moisture of the treated ASR; *V* (mL) is the raw volume of the ASR; $V^{\prime }$ (mL) is the volume of the treated ASR.

#### 2.3.2. Determination of Heavy Metal Content in ASRs and the Stabilization Ratio

The contents of heavy metals such as As, Cd, Cr, Pb, Cu and Zn in the raw material and treated ASR were determined using ICP-AES. Prior to the ICP-AES tests, the sample was digested through microwave-assisted acid digestion according to the procedure described in our previous study [7].

The hydrothermal dehydration process produces a hydrothermal fluid, which results in the loss of some heavy metals. The stabilization ratio of heavy metals was used to evaluate the fixing efficiency of heavy metals in the treated ASR during the treatment, which was calculated according to Equation (3):

(3) $$Stabilization\;ratio=\left(1-\frac{C\times V}{M\times \left(1-\varphi \right)\times w}\right)\times 100\%$$

where *C* (g/mL) is the concentration of heavy metals in the hydrothermal solution after treatment; *V* (mL) is the volume of the hydrothermal solution; *M* (g) is the weight of the raw ASR; *ϕ* (%) is the raw moisture of the ASR; and *w* (%) is the heavy metal content of the raw ASR.

#### 2.3.3. Leaching Test

TCLP tests were performed using the USEPA method to determine the leachability of heavy metals in the samples. The detailed procedure of the TCLP tests was shown in our previous study [32].

#### 2.3.4. Sequential Extraction

A three-step extraction procedure was first proposed by the Community Bureau Reference (BCR). In this work, Davidson’s three-stage BCR sequential extraction procedure was used to analyse the effective combination forms of heavy metals in the residues. The detailed procedure of BCR tests was shown in our previous study [33].

#### 2.3.5. X-ray Photoelectron Spectroscopy (XPS)

X-ray photoelectron spectroscopy (XPS) measurements were carried out on a K-Alpha 1063 system (Thermo Fisher Scientific, Waltham, Massachusetts, USA) using Al-Kα X-ray as the excitation source. The base pressure in the analysis chamber was on the order of 10^−9^ Torr. Peak shifts due to surface charging were taken into account by normalizing energies based on the adventitious carbon peak at 284.5 eV. Survey and narrow-scan XPS spectra were obtained using pass energies of 100 and 30 eV, respectively. Survey scans were used to determine the average composition of the surface. The semi-quantitative composition of the near-surface samples was calculated from the peak areas of the S(2p) and As(3d) peaks and normalized by their respective sensitivity factor [34]. Narrow-scan spectra were obtained in order to determine the S and As surface species.

#### 2.3.6. Others Analysis

The particle size of the raw ASR was analysed in water by a laser particle size analyser (LS-POP (6)). The crystallographic composition of samples was characterized by X-ray diffraction (XRD, D/max2550 VB + 18 KW) at a speed of 10° min^−1^ in a 2θ range from 10° to 80°. Morphological change of the samples was observed through a scanning electron microscope (SEM-EDS, Nova NanoSEM 230, Brno, Czech Republic). Raman spectra (LABRAM-HR 800 spectrometer, Renishaw inVia, Gloucestershire, UK) were recorded with a 513-nm-wavelength He-Le laser and acquisition time of 10 s.

## 3. Results and Discussion

### 3.1. Characterization of the Raw ASR

**Figure 1:**
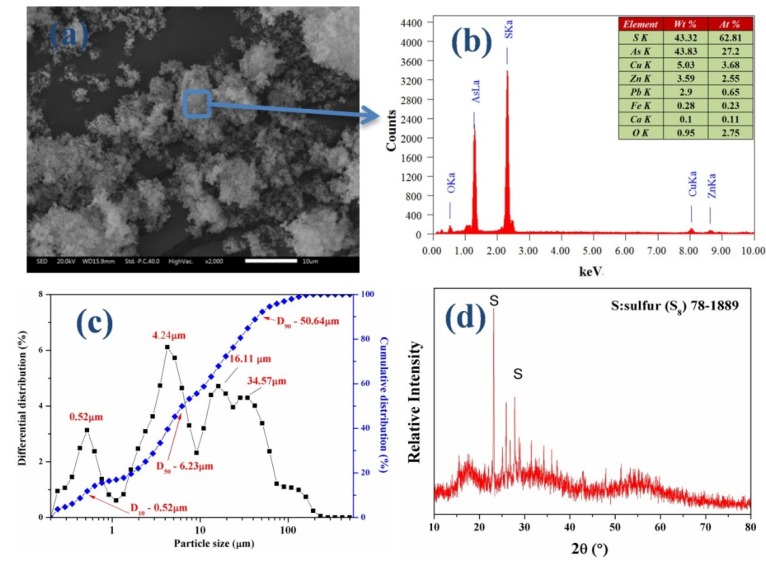
Characterization of the raw HB-ASR: (**a**) SEM; (**b**) EDS; (**c**) Particle size distribution; and (**d**) X-ray diffraction pattern.

The characterization of the raw HB-ASR was selected as a representative to illustrate. The characterization of the raw HB-ASR is presented in Figure 1. As shown in Figure 1a,b,d, the general particles were irregular in shape, and sulfur (S_8_) crystal structures were found. The amorphous particles agglomerate into large particles. The EDS results showed that the residue was mainly composed of S and As, indicating that the amorphous particles are As-S compounds. Figure 1d shows that the three broad peaks occur at 2θ values near 18°, 31° and 57°, similar to that of amorphous As_2_S_3_, as reported [35].

Figure 1c shows a wide range of particle size distribution from 0.5 μm to 60 μm. The size of the particles was distributed in four concentrated areas of approximately 0.52 μm, 4.24 μm, 16.11 μm and 34.57 μm. The median particle size (D_50_) of 6.23 μm indirectly reflected that most small particles were agglomerated into large particles. A leaching test was performed using the TCLP method, and the results indicated that the arsenic-leachate concentration was 300.54 mg/L, which is far greater than the regulation limit of 5 mg/L for As.

### 3.2. Dehydration and Volume Reduction

Table 2 shows the photos of ASRs before and after treatment. In terms of appearance, the yellow muds were transformed into dark-yellow or black blocks. This phenomenon is similar to the result of Gibbs et al. [36] who proposed that the variation in appearance indicated the reduction in band gap resulting from the increase in pressure and temperature. Meanwhile, it is obvious that the muddy or fine particles of ASRs changed to dense smooth solid blocks. Furthermore, the volume shrinkage and dewatering phenomenon can be preliminarily inferred by the appearance changes.

Table 3 lists the moisture, dehydration ratio and volume reduction ratio for each ASR. It is clear that hydrothermal treatment can effectively realize the dehydration and volume reduction for each ASR. The raw ASRs have high contents of moisture, ranging from approximately 39.0% to 62.9%. Nevertheless, the moisture contents for all the treated ASRs were less than 7%. The calculation results of the dehydration ratios show that more than 89% moisture in raw ASRs can be dehydrated via hydrothermal treatment. Moreover, the volume of all ASRs reduced dramatically after hydrothermal treatment. The volume reduction ratios exceeded 60% (HB-ASR 78.67%, SD-ASR 71.42% and AH-ASR 60.10%). The volume reduction ratio is higher when the original ASR contains more water. As a result, the hydrothermal treatment produces excellent dehydration and volume reduction of the ASRs.

### 3.3. Variations in Heavy Metal Contents

Table 4 shows the heavy metal contents in each ASR before and after treatment. After hydrothermal treatment, the contents of As and Cu increased somewhat, whereas the contents of Cd, Cr, Pb and Zn were slightly reduced. This might be attributed to the different behaviors of these heavy metals during the treatment. For example, some adsorbed ions could be washed off, while some sulfides could decompose into soluble salts and volatile H_2_S [37].

Table 5 shows the heavy metals concentration of the hydrothermal solution during the treatment process. Clearly, it can be seen that a large amount of heavy metals were dissolved in the hydrothermal process under high temperature and high pressure, thereby changing the content of heavy metals in the solidified body.

**Figure 2:**
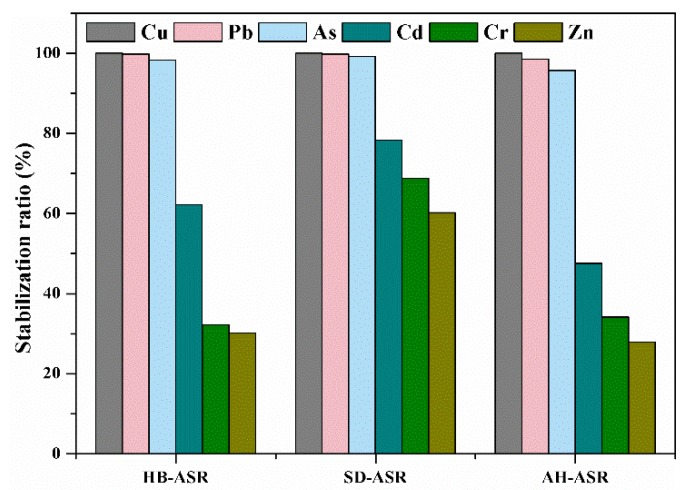
Stabilization ratios of heavy metals for the ASRs.

Since the hydrothermal fluid contained high concentrations of heavy metals, it was necessary to assess the stability of heavy metals in the hydrothermal process and avoid the transfer of contaminants into the wastewater. Figure 2 shows the stabilization ratio for ASRs over the hydrothermal temperature at 160 °C for 4 h. As can be seen from Figure 2, the stabilization ratios of heavy metals from high to low were copper, lead, arsenic, cadmium, chromium and zinc, respectively. The stabilization ratios of arsenic, copper and lead were close to 100%, while most of the chromium and zinc were dissolved in the hydrothermal fluid during the dehydration process. It can be seen that the amount of secondary pollutants produced by the hydrothermal fluid was relatively small, and the impact on the quality of heavy metals in the solidified ASR block is not significant.

### 3.4. Solidification/Stabilization Effect

Table 6 shows the leaching concentrations of the ASRs before and after treatment. The leaching concentrations of As, Cu, Cd, Cr and Zn declined significantly, especially for arsenic. According to the Table 2, the muddy granular ASRs were changed into stabilized/solidified blocks under high temperature/pressure. This was favorable for reducing the leachability of these elements because of the decrease of the specific surface of the ASRs. In addition, some components of heavy metals were probably encapsulated in the densified structure during the condensation process.

### 3.5. Chemical Speciation of Heavy Metals

Three-staged BCR sequential extraction is conducted to assess environmental activity and potential ecological risks. Generally, the arsenic and other heavy metals in acid soluble and reducible fraction is classified as direct effect phases for environmental availability and ecological risk because they are presented as a loosely bound phase or thermodynamically unstable phase, respectively, which are likely to release into the environment. Meanwhile, arsenic associated with the oxidizable fraction is identified as a potential effect fraction because it can be liberated or transformed into an acid soluble and reducible fraction under oxidizing conditions. Only the residual fraction is believed to be a stable fraction because it contains mainly primary and secondary minerals, which may retain metal elements within their crystal or glass structure [29].

The relative percentage of As and other heavy metals extracted in the different steps of the BCR test is presented in Table 7. After the hydrothermal treatment, the acid soluble, reducible and oxidizable soluble fractions of arsenic dramatically decrease from 1.16%, 0.08% and 90.00% to 0.01%, 0.01% and 36.23%, respectively, indicating that direct toxicity effect fractions are reduced [30]. The acid soluble, reducible and oxidizable soluble states of other heavy metals (Cd, Cr, Cu and Zn) also significantly decreased. In terms of residual fractions, the arsenic and other heavy metals (Cd, Cr, Cu, Pb and Zn) of them increased 54.99%, 36.82%, 15.48%, 25.65%, 10.37%, and 43.63%, respectively. Thus, an important conclusion is that the chemical species of arsenic and other heavy metals in sludge are significantly transformed to residual fractions by the hydrothermal treatment, resulting in a restrained environmental availability.

### 3.6. Phase Transformation

**Figure 3:**
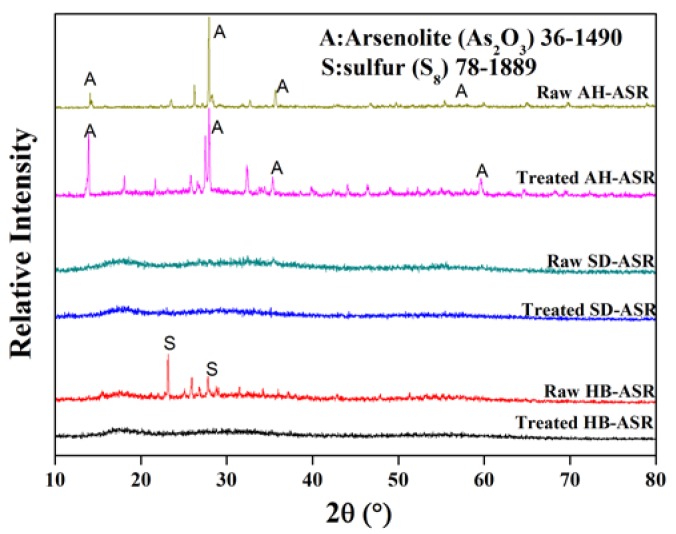
The XRD patterns of the raw and treated ASRs.

Figure 3 shows the XRD patterns of ASRs before and after treatment. As it can be seen from Figure 3, the ASRs before and after treatment were mainly amorphous with only a few diffraction peaks. Only the peaks of arsenic trioxide were found in the raw AH-ASR, probably due to the oxidation of the waste residue during the long-term storage. The crystalline form of sulfur (S_8_) was observed in the raw HB-ASR, which might be attributed to the reaction of S^2−^ and SO_3_^2−^ in the previous waste acid treatment process. No diffraction peaks were found in SD-ASR.

Moreover, for AH-ASR, As_2_O_3_ species were apparently detected in the raw material and the intensity of the As_2_O_3_ peaks increased somewhat after treatment. This could explain the decline in the As leaching concentration as the better crystalline structure meant a lower contact area with the leaching agent. On the other hand, it could be found that the crystalline sulfur (S_8_) in HB-ASR completely disappeared after the treatment. During the hydrothermal process, the sulfur (S_8_) could decompose into H_2_S by disproportionation reaction [38]. The H_2_S gas could precipitate heavy metals and thus reduced their leaching concentrations. For SD-ASR, it was amorphous and no changes on its XRD pattern could be observed after the treatment. Surprisingly, no new crystalline phase of arsenic sulfide compounds, such as realgar and orpiment, were found after hydrothermal treatment. This is probably because under these conditions it was difficult to generate crystalline As_2_S_3_ (c-As_2_S_3_) [39,40]. Although the XRD analysis results can explain the decrease in the leaching concentrations of some metals, it is insufficient and the reason for dehydration and volume reduction may be related to the morphology of the ASRs, which will be discussed in the following section.

### 3.7. Morphology Change

**Figure 4:**
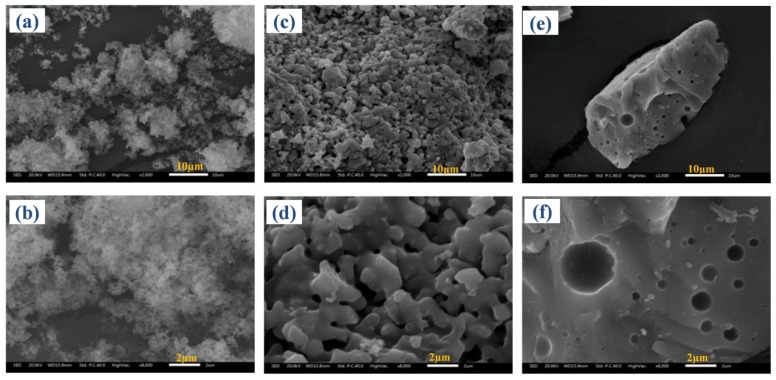
SEM images of raw HB-ASR (**a**,**b**) and treated HB-ASRs (**c**,**d**) treated for 120 min, (**e**,**f**) treated for 240 min).

The SEM images of treated HB-ASR were selected as a representative to illustrate the morphology changes during hydrothermal treatment. Figure 4 shows that there are obvious differences in the morphology of the HB-ASR with different treatment times. Before treatment, the ASR was flocculent particles that were composed of extremely fines of arsenic sulfide (Figure 4a,b). After being treated for 120 min, the flocculent particles bonded together and formed a network of a porous body (Figure 4c,d). In our experiments, it was found that the product generated under this condition was very easily crushed. Finally, a large bulk with a smooth surface was obtained after 240 min (Figure 4e,f). There were some spherular pits on the fracture surface of the bulk, which might be caused byH_2_S gas generated by sulfur decomposition. Because the amorphous and flocculent ASR was converted into a large bulk, the water content and volume of ASR reduced dramatically. This phenomenon was also found in the process of coal treatment by a hydrothermal method [35]. In summary, the densification of ASRs in the hydrothermal process is the main reason for volume reduction, dehydration and stabilization/solidification.

### 3.8. The Analysis of Raman and XPS

**Figure 5:**
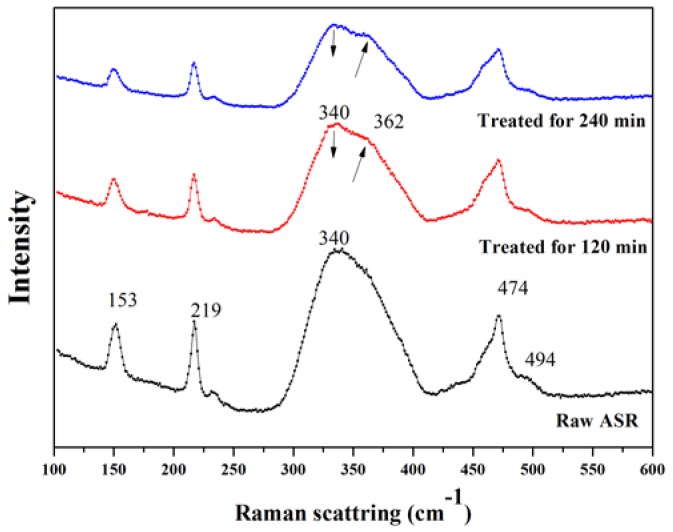
The Raman spectra of the raw and treated HB-ASR.

The Raman technique was further applied to investigate the structural variation in the treated HB-ASR (Figure 5). In the raw ASR, four peaks located at approximately ~153, ~219, ~340, and ~474 cm^−1^ were found. The peak at ~340 cm^−1^ indicated the existence of arsenic(III) sulfide [41,42]. The others (~153, ~219, and ~474 cm^−1^) suggested the presence of sulfur [43]. After the sample was treated for 120 min, the peak at 362 cm^−1^ was found and the intensity became stronger after 240 min, indicating that arsenic(II) sulfide became clearly observable [44,45,46]. The trend of the peak (~340 cm^−1^) shifting to peak (~362 cm^−1^) was probably a reflection of the decomposition of arsenic(III) sulfide into arsenic(II) sulfide.

**Figure 6:**
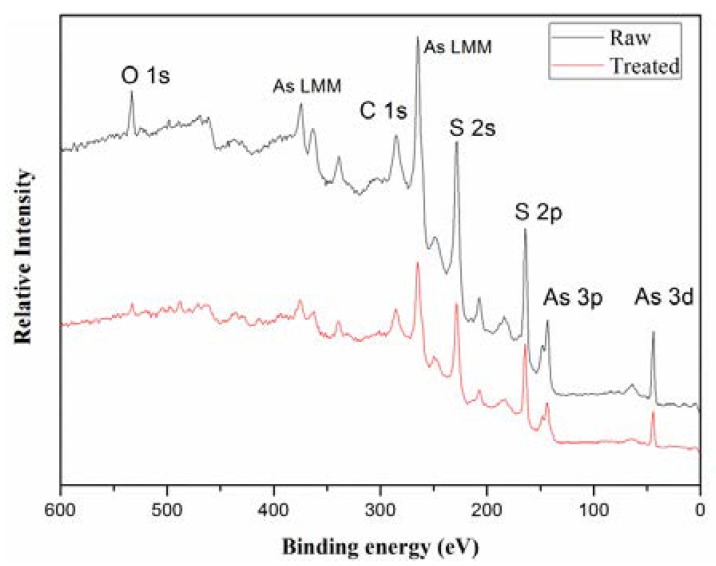
XPS spectra of the raw HB-ASR and that treated for 240 min using a high pass energy of 100 eV.

**Figure 7:**
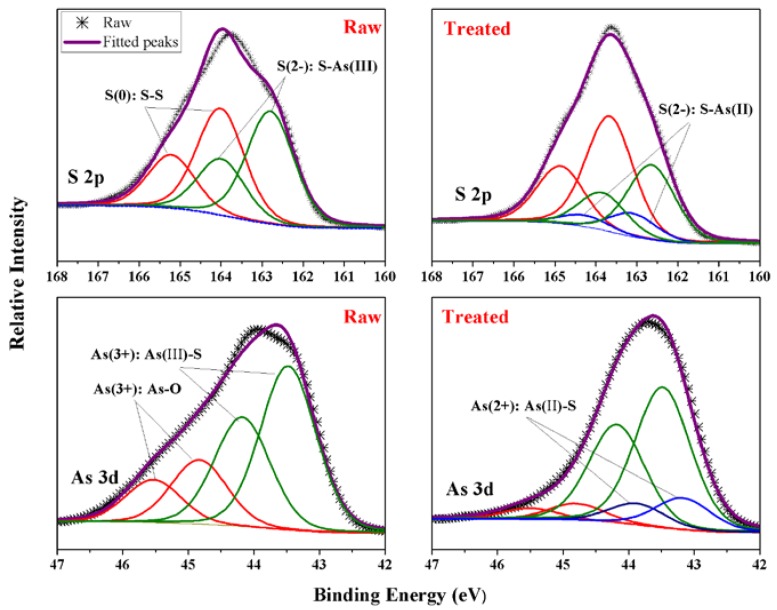
XPS spectra of S 2p and As 3d peaks for the raw HB-ASR and that treated for 240 min using a low pass energy of 30 eV.

XPS survey-spectra of the raw HB-ASR and sample treated for 240 min are shown in Figure 6. It indicates the presence of S, As and O. It was obvious that the intensity of peak O 1s was lower after the treatment. The As 3d and S 2p spectra for the raw and treated sample are presented in Figure 7. The Gaussian-Lorentzian resolving was performed to analyse the components of the sample [47]. Raw spectra were fitted using a least-squares procedure with peaks of convoluted Gaussian (80%) and Lorentzian (20%) peak shape after subtraction of a Shirley baseline. The S 2p spectra were modeled as doublets of 2p_1/2_ and 2p_3/2_, separated by 1.2 eV and the area of the S 2p_1/2_ peak was half the area of S 2p_3/2_ peak. The As 3d spectra were modeled as doublets of 3d_3/2_ and 3d_5/2_, separated by 0.7 eV. The area of the As 3d_3/2_ peak was two-thirds the area of the As 3d_5/2_ peak [48]. A higher binding energy is indicative of a higher oxidation state of arsenic and a lower binding energy corresponds to a lower oxidation state.

The surface compositions of sulfur are shown in Figure 7 and Table 8. The major peaks of the S 2p_3/2_ spectrum of raw ASR were located at 162.80 and 164.02 eV, which were assigned to orpiment-like S^2−^ and S(0), respectively. The reported precipitates of arsenic sulfide formed in the As(III) removing process showed the S 2p_3/2_ binding energies of 162.6 eV and 163.1 eV, which were assigned to the orpiment-like sulfide ion of S-As(III) and realgar-like sulfide ion of S-As(II), respectively [49,50]. As shown in Table 6, after the treatment, the atom ratio of S species increased from 67% to 71%. In addition, the S(0) species accounted for 49% of the total sulfur content in the raw ASR and it increased to 54% after treatment. Finally, it is noted that the realgar-like sulfide ion of S-As(II) also increased to 11%.

The binding energy of the As 3d_5/2_ peak is fitted with three As components consisting of As(III) and As(II). The reported peaks of As 3d_5/2_ at 43.1, 43.4 and 44.8 eV were attributed to As(II)-S, As(III)-S and As(III)-O, respectively [4,27,48,51]. As shown in Table 6, before the treatment, the content of As(III)-S (72%) predominated the As speciation, followed by As(III)-O (28%). However, the content of As(III)-S decreased to 70% and new As(II)-S reached to 17% after the hydrothermal treatment. This change was consistent with results of Raman analysis. On the other hand, Gallegos et al. [52] reported it is thermodynamically favorable for As(III) sulfide to decompose into As_4_S_4_-like phase and S under reducing conditions. Hence, it can be reasoned that the hydrophobic sulfur melts to a liquid to encapsulate the arsenic sulfide compounds, making the particles bond together during the hydrothermal process and thus resulting in the dehydration, volume reduction and S/S of heavy metals.

**Figure 8:**
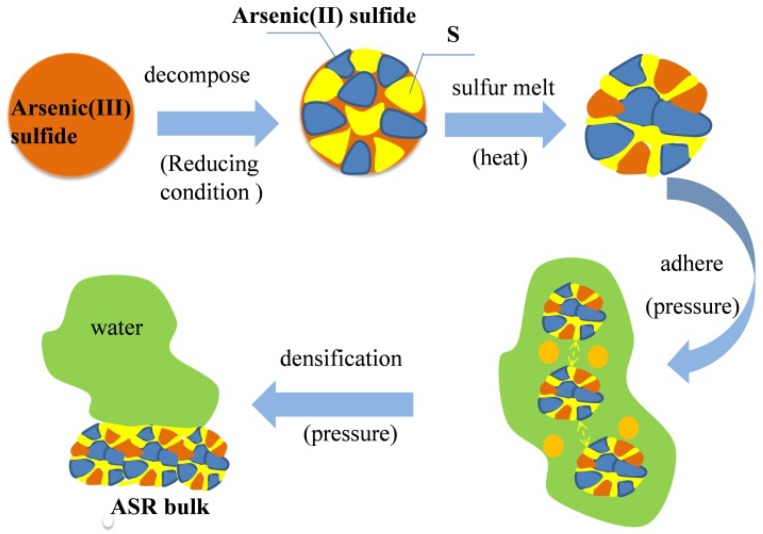
Schematic illustration of the hydrothermal procedure.

Based on the SEM, Raman and XPS results, it is believed that the hydrophobic sulfur (S^0^) and reaction of As(III)-S had a significant influence on the densification of ASR. The schematic diagram for this mechanism is illustrated in Figure 8. First, As(III) sulfide generates hydrophobic sulfur and As(II) sulfide under reductive conditions. Second, the fine particles of As(III) sulfide or As(II) sulfide were bound together by the melted sulfur. Finally, the small formed pieces grew into a larger ASR bulk under high pressure due to adhesion by sulfur. The hydrophobicity of sulfur might be the reason for the satisfactory results of dehydration and volume reduction.

## 4. Conclusions

This study reported the hydrothermal treatment of ASRs for the purpose of dehydration, volume reduction and S/S. The results show that hydrothermal treatment had obvious effects on the dehydration and volume reduction of ASRs. The moisture contents of treated ASR were less than 7% and the dehydration ratios reached 89%~97%. The slurry residues were changed into a hard bulk solid and the volume of the ASRs was reduced by 60%~78%. The stabilization ratios of arsenic, copper and lead were close to 100%, while most of the chromium and zinc was dissolved in the hydrothermal fluid during the dehydration process. After the treatment, the leaching concentrations of As, Cd, Cr and Zn declined significantly and the available arsenic in sludge is significantly transformed to a residual fraction. Based on the further analysis, it was supposed that the As(III) sulfide generates hydrophobic sulfur and As(II) sulfide under reductive conditions. The densification by melting hydrophobic sulfur might play an important role in the dehydration, volume reduction and the decline of heavy metals leaching concentrations. The presented research offers a simple and efficient process for the treatment of ASRs.

However, the evidence from XPS and Raman for the densification mechanism of ASRs is still insufficient. In particular, the action mechanism of action of sulfur requires further deeper analysis and demonstration. Overall, more work has to be done to determine the mechanism of arsenic sulfide and sulfur structural changes during the hydrothermal process and it is also very necessary to perform further tests about its impact on long term landfilling so that the approach can be more reliable and effective.

## Figures and Tables

**Table 1:** The physicochemical properties of the raw ASRs.

Material	Location	Smelting Company	Raw pH	Moisture (%)	As Content (%)
HB-ASR	Hubei	Copper	1.41	62.9	35.1
SD-ASR	Shandong	Zinc-lead	1.22	54.4	34.2
AH-ASR	Anhui	Copper	0.93	39.0	25.7

**Table 2:** Photos of the raw and treated ASRs.

Classification	HB-ASR	SD-ASR	AH-ASR
Before treatment	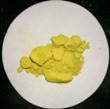	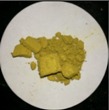	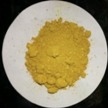
After treatment	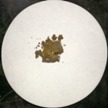	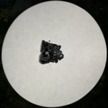	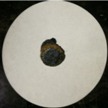

**Table 3:** The comparison of moisture, dehydration ratio and volume reduction ration for each ASR.

Classification		Moisture	Dehydration Ratio	Volume Reduction Ratio
		(wt.%)	(%)	(%)
HB-ASR	Before treatment	62.9	96.80	78.67
	After treatment	4.9		
SD-ASR	Before treatment	54.4	97.79	71.42
	After treatment	2.6		
AH-ASR	Before treatment	39.0	89.74	60.10
	After treatment	6.5		

**Table 4:** Heavy metal contents in the raw and treated ASRs.

Classification		Contents of Heavy Metals (%)					
		As	Cd	Cr	Pb	Cu	Zn
HB-ASR	before treatment	35.1	0.0680	0.0003	0.2600	0.05	0.0820
	after treatment	42.1	0.0230	0.0002	0.2500	0.06	0.0110
SD-ASR	before treatment	34.2	0.0011	0.0002	0.0660	0.31	0.0012
	after treatment	37.7	0.0009	0.0001	0.0820	0.38	0.0011
AH-ASR	before treatment	25.7	0.0056	0.0032	0.0044	0.71	0.0110
	after treatment	43.0	0.0016	0.0010	0.0042	1.13	0.0026

**Table 5:** Concentration of heavy metals in the hydrothermal fluid of treated ASRs.

Sample	V (mL)	As	Cd	Cr	Pb	Cu	Zn
		(mg/L)					
HB-ASR	6.1	3437.0	151.5	1.20	2.50	0.03	337.50
SD-ASR	5.3	2302.5	2.0	0.53	0.98	0.90	4.00
AH-ASR	3.5	17,160.0	46.0	33.00	1.00	0.04	124.00

**Table 6:** Leaching concentration of heavy metals in the raw and treated ASRs.

Classification		Leaching Concentration of Heavy Metals (mg/L)					
		As	Cd	Cr	Pb	Cu	Zn
HB-ASR	before treatment	300.54	11.20	0.13	2.45	0.05	0.08
	after treatment	1.68	0.05	ND	1.49	0.01	0.03
SD-ASR	before treatment	39.12	0.08	0.03	0.07	0.31	0.02
	after treatment	1.11	0.02	ND	0.09	0.13	0.01
AH-ASR	before treatment	3860.25	2.45	1.62	1.38	21.43	5.61
	after treatment	126.30	0.15	0.66	0.53	0.32	0.64

**Table 7:** Chemical speciation of arsenic and heavy metals by BCR procedure (wt.%) in the raw and treated HB-ASR.

Classification		As	Cd	Cr	Cu	Pb	Zn
Before treatment	Acid soluble	1.16	17.59	12.61	0.49	1.12	22.96
	Reducible	0.08	1.96	2.64	0.24	1.12	1.71
	Oxidizable	90.00	70.37	18.3	70.49	63.43	51.25
	Residual	8.76	10.08	66.45	28.78	34.33	24.08
After treatment	Acid soluble	0.01	0.40	1.66	0	19.49	1.03
	Reducible	0.01	0	0	0	7.21	0.07
	Oxidizable	36.23	51.98	16.41	45.57	28.6	31.19
	Residual	63.75	47.62	81.93	54.43	44.7	67.71

**Table 8:** Peak areas for component peaks used in fitting the As (3d) and S (2p) peaks for the raw HB-ASR and treated ASRs.

Bending Energy and Percent Peak Area of Each Component									
Sample		As 3d	S 2p	As 3d5/2			S 2p3/2		
		As	S	As(II)-S	As(III)-S	As(III)-O	S-S	S-As(III)	S-As(II)
Raw ASR	BE (FWHM)	43.83 (2.03)	163.73 (2.86)	43.10 (1.00)	43.48 (1.00)	44.80 (1.00)	164.02 (1.37)	162.80 (1.37)	163.10 (1.37)
	Peak areas	33%	67%	0	72%	28%	49%	51%	0
Treated ASR	BE (FWHM)	43.69 (1.61)	163.58 (2.47)	43.28 (1.00)	43.42 (1.00)	44.50 (1.00)	163.66 (1.37)	162.64 (1.37)	163.10 (1.37)
	Peak areas	29%	71%	17%	70%	13%	54%	35%	11%

BE: Bending energy (FWHM); Narrow-scan XPS spectra were obtained using pass energies of 30 eV.

## References

[1] Am S.D.L.C., Sánchez-Rodas D., González C.Y., Jd D.L.R. (2015). Geochemical anomalies of toxic elements and arsenic speciation in airborne particles from Cu mining and smelting activities: Influence on air quality. J. Hazard. Mater..

[2] Zhang Q.L., Gao N.Y., Lin Y.C., Xu B., Le L.S. (2007). Removal of Arsenic(V) from Aqueous Solutions Using Iron-Oxide-Coated Modified Activated Carbon. Water Environ. Res..

[3] Chai L.Y., Yang J.Q., Zhang N., Wu P.J., Li Q.Z., Wang Q.W., Liu H., Yi H.B. (2017). Structure and spectroscopic study of aqueous Fe(III)-As(V) complexes using UV-Vis, XAS and DFT-TDDFT. Chemosphere.

[4] Fei J.C., Wang T., Zhou Y.Y., Wang Z.X., Min X.B., Ke Y., Hu W.Y., Chai L.Y. (2018). Aromatic organoarsenic compounds (ADCs) occurrence and remediation methods. Chemosphere.

[5] Fei J.C., Min X.B., Wang Z.X., Pang Z.H., Liang Y.J., Ke Y. (2017). Health and ecological risk assessment of heavy metals pollution in an antimony mining region: A case study from South China. Environ. Sci. Pollut. Res..

[6] Luo T., Cui J.L., Hu S., Huang Y.Y., Jing C.Y. (2010). Arsenic Removal and recovery from copper smelting wastewater using TiO_2_. Environ. Sci. Technol..

[7] Ke Y., Shen C., Min X.B., Shi M.Q., Chai L.Y. (2017). Separation of Cu and As in Cu-As-containing filter cakes by Cu^2+^-assisted acid leaching. Hydrometallurgy.

[8] Peng Y.L., Zheng Y.J., Chen W.M. (2012). The oxidation of arsenic from As(III) to As(V) during copper electrorefining. Hydrometallurgy.

[9] Zheng Y.J., Peng Y.L., Ke L., Chen W.M. (2013). Separation and recovery of Cu and As from copper electrolyte through electrowinning and SO_2_ reduction. Trans. Nonferrous Metal. Soc..

[10] Andjelkovic I., Jovic B., Jovic M., Markovic M., Stankovic D., Manojlovic D., Roglic G. (2016). Microwave-hydrothermal method for the synthesis of composite materials for removal of arsenic from water. Environ. Sci. Pollut. Res..

[11] Min X., Li Y., Ke Y., Shi M., Chai L., Xue K. (2017). Fe-FeS_2_ adsorbent prepared with iron powder and pyrite by facile ball milling and its application for arsenic removal. Water Sci. Technol..

[12] Peng C., Chai L.Y., Tang C.J., Min X.B., Song Y.X., Duan C.S., Yu C. (2017). Study on the mechanism of copper-ammonia complex decomposition in struvite formation process and enhanced ammonia and copper removal. J. Environ. Sci. China.

[13] Chai L.Y., Yue M.Q., Yang J.Q., Wang Q.W., Li Q.Z., Liu H. (2016). Formation of tooeleite and the role of direct removal of As(III) from high-arsenic acid wastewater. J. Hazard. Mater..

[14] Gimenez-Forcada E., Vega-Alegre M., Timon-Sanchez S. (2017). Characterization of regional cold-hydrothermal inflows enriched in arsenic and associated trace-elements in the southern part of the Duero Basin (Spain), by multivariate statistical analysis. Sci. Total Environ..

[15] Bai M., Zheng Y.J., Liu W.Y., Zhang C.F. (2008). Alkaline leaching and leaching kinetics of arsenic sulfide residue. J. Cent. South Univ..

[16] Yang B., Zhang G.L., Deng W., Ma J. (2013). Review of Arsenic Pollution and Treatment Progress in Nonferrous Metallurgy Industry. Adv. Mater. Res..

[17] Hu H.J., Qiu K.Q. (2015). Three-step vacuum separation for treating arsenic sulphide residue. Vacuum.

[18] Li Y.C., Min X.B., Chai L.Y., Shi M.Q., Tang C.J., Wang Q.W., Liang Y.J., Lei J., Liyang W.J. (2016). Co-treatment of gypsum sludge and Pb/Zn smelting slag for the solidification of sludge containing arsenic and heavy metals. J. Environ. Manag..

[19] Liu D.G., Min X.B., Ke Y., Chai L.Y., Liang Y.J., Li Y.C., Yao L.W., Wang Z.B. (2018). Co-treatment of flotation waste, neutralization sludge, and arsenic-containing gypsum sludge from copper smelting: Solidification/stabilization of arsenic and heavy metals with minimal cement clinker. Environ. Sci. Pollut. Res..

[20] Lei J., Peng B., Min X.B., Liang Y.J., You Y., Chai L.Y. (2017). Modeling and optimization of lime-based stabilization in high alkaline arsenic-bearing sludges with a central composite design. J. Environ. Sci. Health A.

[21] Lei J., Peng B., Liang Y.J., Min X.B., Chai L.Y., Ke Y., You Y. (2018). Effects of anions on calcium arsenate crystalline structure and arsenic stability. Hydrometallurgy.

[22] Zhou S., Shang T., Zhong P., Liu W. (2015). Stabilization and solidification of strong acidic arsenic sulfide residue. Environ. Proc. Chem. Ind..

[23] Du Y., Xiao H., Lu Q., Du D. (2017). Using manganese slag to stabilize/solidify arsenic sulfide slag by moderate temperature calcination. Chin. J. Environ. Eng..

[24] Chai L.Y., Yue M.Q., Li Q.Z., Zhang G.S., Zhang M.X., Wang Q.W., Liu H., Liu Q.W. (2018). Enhanced stability of tooeleite by hydrothermal method for the fixation of arsenite. Hydrometallurgy.

[25] Vinals J., Sunyer A., Molera P., Cruells M., Llorca N. (2010). Arsenic stabilization of calcium arsenate waste by hydrothermal precipitation of arsenical natroalunite. Hydrometallurgy.

[26] Qiu Q.L., Jiang X.G., Lv G.J., Lu S.Y., Ni M.J. (2016). Stabilization of heavy metals in municipal solid waste incineration fly ash in circulating fluidized bed by microwave-assisted hydrothermal treatment with additives. Energy Fuel.

[27] Chai L.Y., Ke Y., Min X.B., Zhou B.S., Xue K., Chen J. (2015). Separation and recovery of ZnS from sulfidized neutralization sludge via the hydration conversion of CaSO_4_ into bulk CaSO_4_·2H_2_O crystals. Sep. Purif. Technol..

[28] Ke Y., Chai L.Y., Min X.B., Tang C.J., Zhou B.S., Chen J., Yuan C.Y. (2015). Behavior and effect of calcium during hydrothermal sulfidation and flotation of zinc-calcium-based neutralization sludge. Miner. Eng..

[29] Liang Y.J., Min X.B., Chai L.Y., Wang M., Liyang W.J., Pan Q.L., Okido M. (2017). Stabilization of arsenic sludge with mechanochemically modified zero valent iron. Chemosphere.

[30] Xie X.D., Min X.B., Chai L.Y., Tang C.J., Liang Y.J., Li M., Ke Y., Chen J., Wang Y. (2013). Quantitative evaluation of environmental risks of flotation tailings from hydrothermal sulfidation-flotation process. Environ. Sci. Pollut. Res..

[31] Theodore I., King I.I. (1993). The Effect of water temperature on hand volume during volumetric measurement using the water displacement method. J. Hand Ther..

[32] Ke Y., Chai L.Y., Min X.B., Tang C.J., Chen J., Wang Y., Liang Y.J. (2014). Sulfidation of heavy-metal-containing neutralization sludge using zinc leaching residue as the sulfur source for metal recovery and stabilization. Miner. Eng..

[33] Min X.B., Xie X.D., Chai L.Y., Liang Y.J., Mi L.I., Yong K.E. (2013). Environmental availability and ecological risk assessment of heavy metals in zinc leaching residue. Trans. Nonferrous Met. Soc..

[34] Renock D., Gallegos T., Utsunomiya S., Hayes K., Ewing R.C., Becker U. (2009). Chemical and structural characterization of As immobilization by nanoparticles of mackinawite (FeSm). Chem. Geol..

[35] Kong L.H., Peng X.J., Hu X.Y. (2017). Mechanisms of UV-Light Promoted removal of As(V) by sulfide from strongly acidic wastewater. Environ. Sci. Technol..

[36] Gibbs G.V., Wallace A.F., Zallen R., Downs R.T., Ross N.L., Cox D.F., Rosso K.M. (2010). Bond paths and van der waals interactions in orpiment, As_2_S_3_. J. Phys. Chem A.

[37] Yuichi T., Naoki H., Masami T. (2006). Removal of arsenic ions in sulfuric acid solutions by sulfide minerals. Min. Mater. Process. Inst. Jpn..

[38] Bottcher M.E., Thamdrup B., Vennemann T.W. (2001). Oxygen and sulfur isotope fractionation during anaerobic bacterial disproportionation of elemental sulfur. Geochim. Cosmochim. Acta.

[39] Cernosek Z., Cernoskova E., Benes L. (1999). Crystalline arsenic trisulfide: Preparation, differential scanning calorimetry and Raman scattering measurements. Mater. Lett..

[40] Yang C.Y., Paesler M.A., Sayers D.E. (1986). First crystallization of arsenic trisulfide from bulk glass: The synthesis of orpiment. Mater. Lett..

[41] Rochette E.A., Bostick B.C., Li G.C., Fendorf S. (2000). Kinetics of arsenate reduction by dissolved sulfide. Environ. Sci. Technol..

[42] Arsova D., Boulmetis Y.C., Raptis C., Pamukchieva V., Skordeva E. (2005). The boson peak in Raman spectra of As_x_S_1-x_ glasses. Semiconductors.

[43] Godelitsas A., Price R.E., Pichler T., Amend J., Gamaletsos P., Gottlicher J. (2015). Amorphous As-sulfide precipitates from the shallow-water hydrothermal vents off Milos Island (Greece). Mar. Chem..

[44] Shpotyuk O., Kovalskiy A., Trimble J., Vicek M., Shpotyuk Y., Kozyukhin S. (2015). Intrinsic phase separation in low-temperature quenched arsenic trisulfide glass. J. Non-Cryst. Solids.

[45] Shastry M.C.R., Couzi M., Levasseur A., Ménétrier M. (1993). Raman spectroscopic studies of As_2_S_3_ and Li_2_S–As_2_S_3_ glasses. Philos. Mag. Part B.

[46] Slade M.L., Zallen R. (1979). Raman spectra of As_4_S_4_ polymorphs: Structural implications for amorphous As_2_S_3_ films. Solid State Commun..

[47] Synowicki R.A., Tiwald T.E. (2004). Optical properties of bulk c-ZrO_2_, c-MgO and a-As_2_S_3_ determined by variable angle spectroscopic ellipsometry. Thin Solid Films.

[48] Kim E.J., Batchelor B. (2009). Macroscopic and X-ray Photoelectron Spectroscopic Investigation of Interactions of Arsenic with Synthesized Pyrite. Environ. Sci. Technol..

[49] Hu H.Y., Fang Y., Liu H., Yu R., Luo G.Q., Liu W.Q., Li A.J., Yao H. (2014). The fate of sulfur during rapid pyrolysis of scrap tires. Chemosphere.

[50] Liu R.P., Yang Z.C., He Z.L., Wu L.Y., Hu C.Z., Wu W.Z., Qu J.H. (2016). Treatment of strongly acidic wastewater with high arsenic concentrations by ferrous sulfide (FeS): Inhibitive effects of S(0)-enriched surfaces. Chem. Eng. J..

[51] Stec W.J., Morgan W.E., Albridge R.G., Wazer J.R.V. (2002). Measured binding energy shifts of “3p” and “3d” electrons in arsenic compounds. Inorg. Chem..

[52] Gallegos T.J., Han Y.S., Hayes K.F. (2008). Model Predictions of Realgar Precipitation by Reaction of As(III) with Synthetic Mackinawite Under Anoxic Conditions. Environ. Sci. Technol..

